# Can using a resource use log in an economic evaluation alongside a randomised controlled trial reduce the amount of recall bias?

**DOI:** 10.1186/1745-6215-16-S2-O25

**Published:** 2015-11-16

**Authors:** Sian Noble, Isobel Tudge, Vikki Wylde, Erik Lenguerrand, Elsa Marques

**Affiliations:** 1 University of Bristol, Bristol, UK

## Objective

To determine whether giving patients a resource use log (RUL) at hospital discharge reduces recall bias in a follow-up resource use questionnaire (RUQ).

## Methods

85 patients undergoing joint replacement were randomised to receive or not receive an RUL at hospital discharge. A postal RUQ was then administered to participants at 3-months after surgery.

A blinded researcher extracted primary care resource use data in relation to the patient's joint replacement from GP records from hospital discharge until completion of the 3-month RUQ. Data from both sources were coded into use of resource and number of contacts and two binary variables indicating perfect recall were calculated. For each resource use category, descriptive statistics were calculated by data source and trial arm, and adjusted odds ratios were estimated.

## Results

GPs were contacted for 67/85 patients originally randomised (3 died, 4 withdrew, 4 did not return the 3-month RUQ, 6 had GP practices outside of area). Information was extracted for 66/67 patients.

Those in the ‘RUL’ arm, with the exception of “Phoned GP for advice” (use of a resource and number of contacts) and “GP nurse visit” (number of contacts) had more agreement between the data sources.

Patients in the RUL arm had greater adjusted odds 9.77 (95%C.I. 1.69, 56.31) of recalling whether they had a GP surgery visit.

## Discussion

Although underpowered, our study found some evidence that provision of an RUL improves recall and indicates that an RUL is a useful tool when conducting within-trial economic evaluations.

